# Efficacy and safety of ERCP in patients with situs inversus totalis: multicenter case series and literature review

**DOI:** 10.1186/s12876-022-02593-3

**Published:** 2022-11-30

**Authors:** Bin Ding, Jun Wang, Xing Wei, Yu Du, Liang Xia, Chenyi Sun, Kun Han, Xue Yang, Xuegang Guo, Yanglin Pan, Xiangping Wang

**Affiliations:** 1 Department of Gastroenterology, Jiujiang No.1 People’s Hospital, Jiujiang, China; 2 Department of Gastroenterology, Fourth Military Medical University, No. 986 Hospital, Xi’an, China; 3 Department of Gastroenterology, Yuncheng Central Hospital, Shanxi Medical University, Yuncheng, China; 4 Department of Gastroenterology, The Second People’s Hospital of Qinzhou, Qinzhou, China; 5 Department of Gastroenterology, The First Affiliated Hospital of Nanchang University, Nanchang, China; 6 Department of Gastroenterology, The Second Affiliated Hospital of Zhengzhou University, Zhengzhou, China; 7 Department of Gastroenterology, Xi’an Central Hospital, Xi’an, China; 8 Department of Hepatobiliary Surgery, The First Affiliated Hospital of Xi’an Jiaotong University, Xi’an, China; 9 National Clinical Research Center for Digestive Diseases and Xijing Hospital of Digestive Diseases, Fourth Military Medical University, 127 Changle West Road, Xi’an, 710032 Shaanxi China

**Keywords:** ERCP, Situs inversus totalis, Cannulation, Complication, Patient position

## Abstract

**Background:**

Endoscopic retrograde cholangiopancreatography (ERCP) in patients with situs inversus totalis (SIT) is rarely understood due to its rarity. Patient position and endoscope manipulation were the main concerns in published case reports. The aim of this study was to investigate the efficacy and safety of ERCP in SIT patients.

**Methods:**

Patients with SIT who underwent ERCP were enrolled in nine endoscopic centers in China. ERCP procedural details and complications in SIT patients were retrieved from electronic medical record. The data was retrospectively analyzed.

**Results:**

From 2011 to 2021, totally 14 patients with SIT undergoing ERCP were identified. The mean age was 56.8 years old and the male–female ratio was 5:2. The main indication for ERCP was common bile duct stones (13/14, 92.9%). All procedure were performed by experienced endoscopists. 21.4% (3/14) of patients were under regular position (prone), while 78.6% under modified position (supine, left or right lateral). Difficult cannulation was occurred in 71.4% (10/14) of patients. The rate of successful cannulation was 85.7% (12/14). Complication occurred in 3 patients (3/14, 21.4%), including 1 bleeding, 1 pneumonia and 1 acute myocardial infarction. No post-pancreatitis or death happened. Compared to patients in modified position, those in prone position had numerically less successful cannulations (66.7% vs. 90.9%) and higher adverse events (33.3% vs. 18.2%).

**Conclusions:**

ERCP in patient with SIT is challenging even for experienced endoscopists, modified patient positions might have potential benefits concerning more successful cannulations and less complications. More case experiences are need for comprehensive understanding of ERCP in patients with SIT.

**Supplementary Information:**

The online version contains supplementary material available at 10.1186/s12876-022-02593-3.

## Background

Situs inversus totalis (SIT) is a rare congenital condition, with an incidence of approximately 1 in 5000–20,000, which is usually asymptomatic but poses a challenge to surgical and endoscopic intervention of asymmetric organs due to its characterized mirror-image transposition of all viscera [1]. Laparoscopic procedure of SIT patients is more inconvenient and time-consuming for right-handed surgeon, requiring modified placement of operating ports/trocars and surgical teams [2]. Inversely position alteration and maneuver modification are also warranted during gastrointestinal endoscopic procedure to facilitate lesion exposure and dissection [1, 3, 4].

As a technically demanding and dangerous gastrointestinal endoscopic procedure, ERCP is considered even more difficult in SIT patients [5]. The left–right coordination is often highly required for selective cannulation. Challenges for endoscopists also lie in adapting inversed endoscopic image, fluoroscopic image, and cannulation direction simultaneously. Studies on this issue are less than 50 in the PubMed to date because of its rarity. They were all sporadic case reports, presenting variation on endoscope manipulation and patient-operator position [6–9]. However, optimal technique and patient-endoscopist position during ERCP in SIT patients are still unclear. Its impacts on ERCP procedure and complication are underinvestigated.

In this study we aimed to report our multicenter experience with the efficacy and safety of ERCP in SIT patients. All related cases reported in the literature are also summarized.

## Methods

This multi-center retrospective cohort study was conducted at Jiujiang Hospital and other eight tertiary endoscopy centers in China. Electronic medical record was searched for patients undergoing ERCP at each center between 2011 and 2021. The study protocol was approved by institutional review board of all hospitals. All authors had access to the study data and reviewed and approved the final article.

### Patients

Consecutive patients aged 18–85 years old who underwent ERCP and proved SIT by computed tomography (CT) or magnetic resonance imaging (MRI) were eligible. The exclusion criteria included: 1, intubation failure or unable to find the major papilla; 2, prior surgery of gastrointestinal reconstruction; 3, severe organ dysfunction; 4, pregnant or lactating women.

### ERCP procedure

ERCP was performed under propofol/diazepam plus piperidine sedation or general anesthesia. Position of patients and endoscopists were decided at the discretion of the endoscopists and anesthesiologists. Endoscopic and fluoroscopic monitors were customarily placed on the patient’s head side. When patient was placed in the prone, supine or left lateral position, the endoscopist stood at the left side of table as usual. For patients placed in the supine position, extra position adjustments were made to avoid respiratory complications, including raised the patient’s right shoulder with pillow or chest roll and turned patient’s head to the left, kept a head up and feet down position. Intermittent salivary aspiration and intensive monitoring were also applied to those patients. The duodenoscope was counterclockwise rotated 180° in stomach and the second part of duodenum in prone or left lateral position, the biliary cannulation was pointed to the 1–3 o’clock direction of papilla orifice. When patient was placed in the right lateral position, endoscopist stood at the right side of the table, “mirror image” technique was used for scope intubation and selective cannulation as described by García-Fernández FJ et al. [7]. All procedures were performed with standard duodenoscope and accessories. Wire-guided cannulation was used as the first-line method for selective cannulation. Double-wire technique (DWT) or precut (transpancreaitc or free-hand) was performed when standard cannulation failed. Balloon or basket were used for stones retrieval, plastic stent or naso-biliary drainage was placed if complete stone clearance could not be confirmed. The type of stent was at the choice of endoscopists. Prophylactic pancreatic duct stenting or rectal indomethacin were used in patients who were at high risk for post-ERCP pancreatitis (PEP). All the participating endoscopists were experienced and had performed at least 1000 ERCP independently.

### Data collection and follow-up

Demographic data, clinical data, radiological imaging and ERCP procedure-related data were retrospectively collected. SIT was diagnosed according to imaging interpretation of MRI or CT before ERCP. Difficult cannulation was considered when total cannulation time was more than 5 min, the total cannulation attempts were more than 5 times or there was more than one inadvertent pancreatic duct cannulation [10]. Successful cannulation was defined as deep cannulation of targeted duct. Technical success was defined as complete stone clearance or appropriate stenting. Procedure time was calculated from oral insertion of duodenoscope to the withdraw of the scope. High risk for PEP was defined based on criteria used in the study of Luo and colleagues [11] (Additional file 1: Table S1). Complications were defined and classified according to the criteria of Cotton and the revised Atlanta criteria [12, 13]. Patients were followed up until December 2021.

### Statistical analysis

Quantitative variables were expressed as means and standard deviation (SD), or medians and interquartile range (IQR). Categorical variables were expressed as frequencies or percentages. Data analyses were performed with IBM SPSS (version 26.0) or Excel.

## Results

### Baseline characteristics

**Fig. 1:**
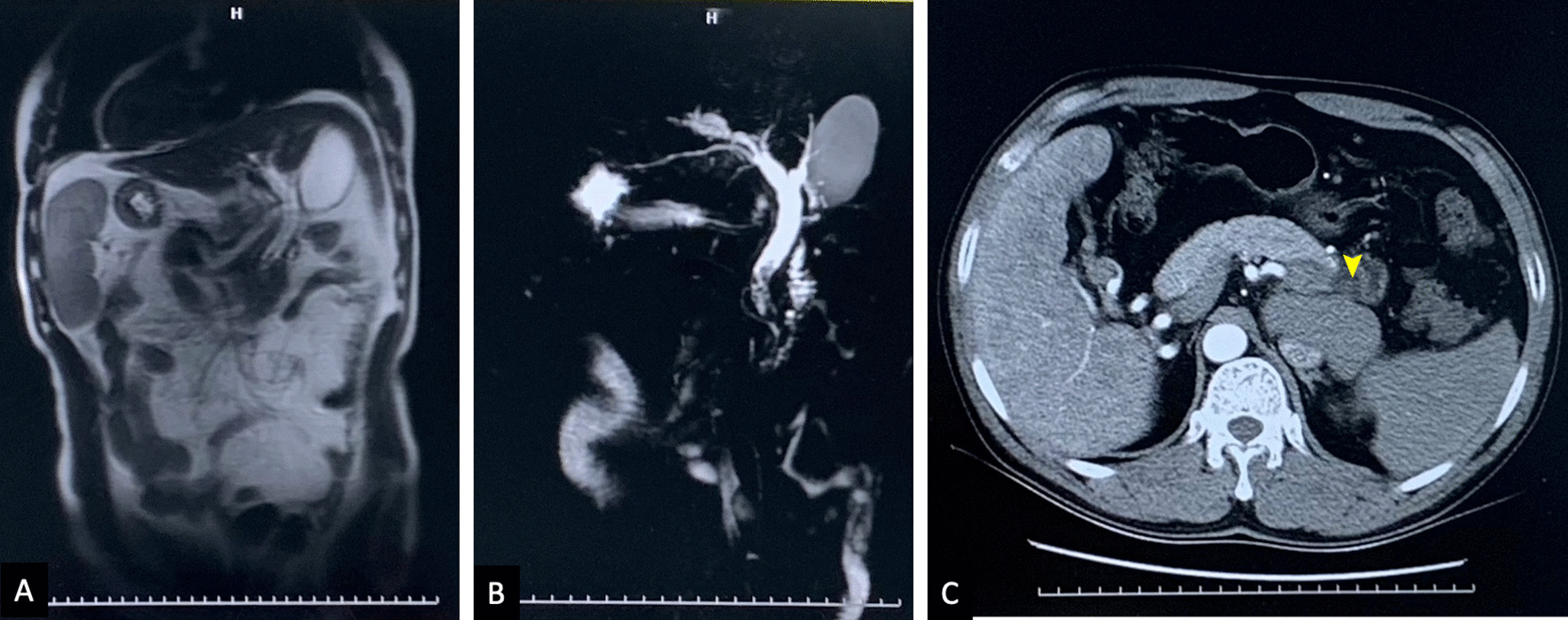
MRI/MRCP and CT images of a SIT patient with CBDS and decompensated cirrhosis. **A**. coronal MRI image shows the mirror-image transposition of abdominal and thorax viscera. **B**. reconstruction image indicates multiple small stones piled inside the middle-distal of CBD, the conference of bile duct and pancreatic duct seems located in the lower corner or the horizontal part of duodenum. **C**. CT image of the SIT patients, with yellow arrowhead points to the hypodense or isodense biliary stones

From January 2011 to December 2021, 65,838 patients had undergone ERCP in nine centers and were screened for the study. 14 (0.02%) with SIT were included (Table 1). All of them had native papilla. The mean age of the study population at ERCP was 56.8 years old. 10 (71.4%) of the patients were male. SIT were diagnosed by CT and MRI in all patients (Fig. 1). None was found with concomitant congenital abnormality. The indication of ERCP included common bile duct stones (CBDS) (92.9%, 13/14) and suspected biliary acute pancreatitis (7.1%, 1/14). 5 (35.7%) patients had at least one comorbidity. 8 (57.1%) patients had at least one stone with a maximum diameter ≥ 10 mm.

**Table 1:** Demographic characteristics of the 14 SIT patients underwent ERCP

	Patient (n = 14)
Age, median(IQR)	52.5 (40.8–72.5)
Age, mean ± SD	56.8 ± 18.4
Male, n (%)	10 (71.4)
ERCP indication, n (%)	
CBDS	13 (92.9)
Acute cholangitis	8 (57.1)
Biliary acute pancreatitis	1 (7.1)
Comorbidity, n (%)	5 (35.7)
Coronary heart disease	4 (28.6)
Hypertension	3 (21.4)
Type 2 diabetes mellitus	1 (7.1)
Cirrhosis	1 (7.1)
Chronic obstructive pulmonary disease	2 (14.3)

*CBDS*, common bile duct stone; *ERCP*, endoscopic retrograde cholangiopancreatography; *SIT*, situs versus totalis

### ERCP procedure

Patients were initially placed in supine (35.7%, 5/14), left lateral (28.6%, 4/14), prone (21.4%, 3/14) and right lateral (14.3%, 2/14) position, as listed in Table 2. 10 endoscopists performed ERCP on the left side of the table while the left 2 on the right side. 2 patients had position change during cannulation, including one from supine to prone and the other from right lateral to supine.

**Table 2:** ERCP procedural details of the 14 SIT patients

	Patient (n = 14)
*Patient position(initial)*	
Prone, n (%)	3 (21.4%)
Supine, n (%)	5 (35.7%)
Left lateral, n (%)	4 (28.6%)
Right lateral, n (%)	2 (14.3%)
Position change, n (%)	2 (14.3%)
*Sedation, n (%)*	
General anesthesia	2 (14.3%)
Conscious sedation	12 (85.7%)
Ectopic papilla, n (%)	1 (7.1%)
PAD, n (%)	1 (7.1%)
Intubation success, n (%)	14 (100)
*Cannulation method*	
Wire-guided, n (%)	13 (92.9%)
Needle knife, n (%)	1 (7.1%)
Transpancreatic precut, n (%)	2 (14.3%)
Difficult cannulation, n (%)	10 (71.4)
Cannulation success, n (%)	12 (85.7%)
Technical success, n (%)	12 (85.7%)
Procedure time (min), median(IQR)	45 (27.5–60)

*ERCP*, endoscopic retrograde cholangiopancreatography; *PAD*, periampullary diverticulum

Papilla of all patients were successfully reached and brought to appropriate direction by adjustment of scope tip or patient position if necessary. Long scope position was needed during procedure in 3 patients. Type A periampullary diverticulum (PAD) was identified in 1 patient. 1 patient had ectopic papilla distally located in the lower duodenal angle.

**Fig. 2:**
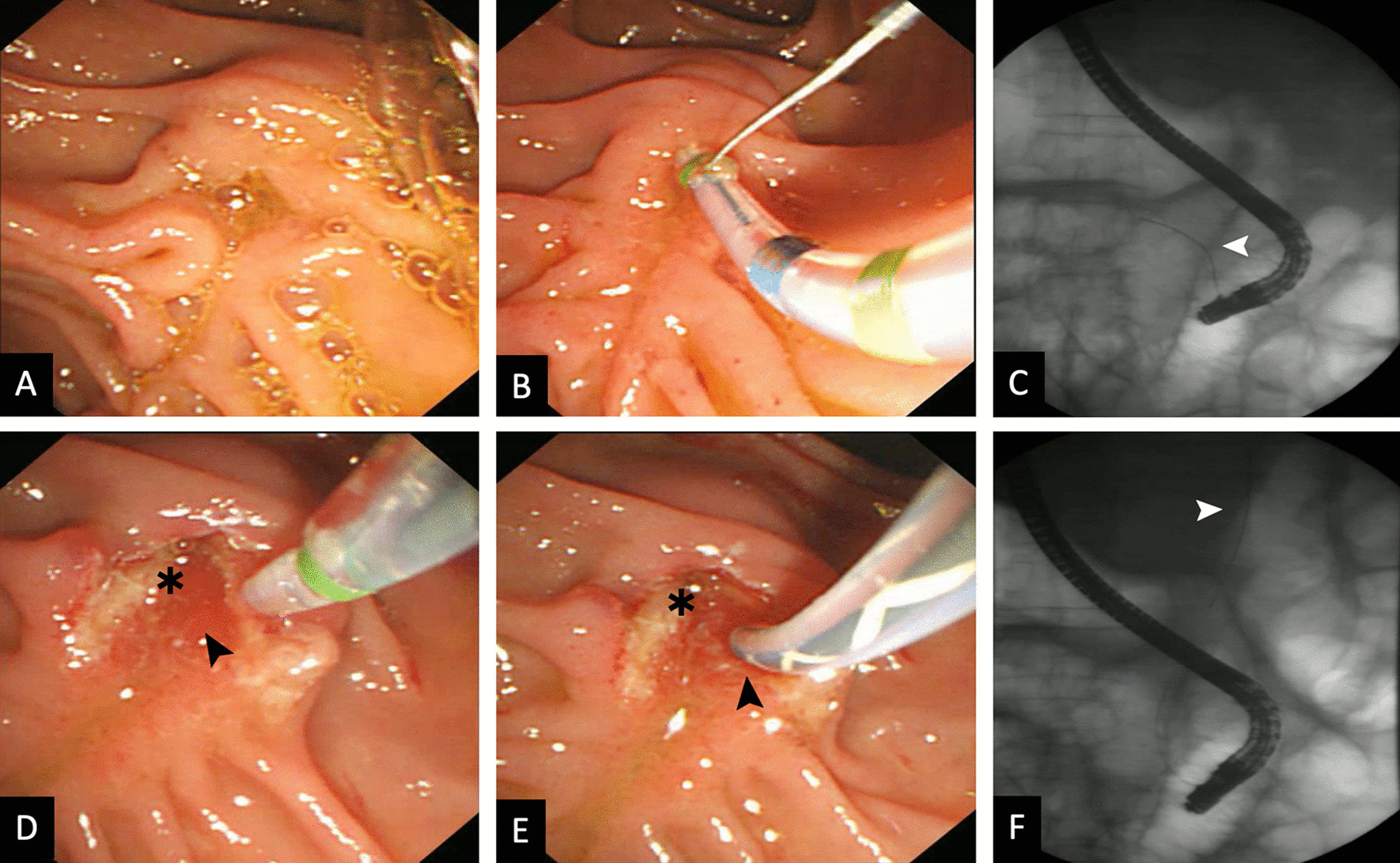
Different biliary cannulation direction of a SIT patient under prone position(the same patient in Fig. 1). Endoscopic view showed that the papilla was in the right side with a longitudinal axis of 2 o’clock (**A)**. Initial cannulation direction aiming to 11 o’clock (**B**) resulted in inadvertent pancreatic duct cannulation (**C**). Fluoroscopy showed guidewire was in the pancreatic duct. The biliary orifice (black arrowhead) at 3 o’clock was then exposed after transpancreatic precut, while the pancreatic orifice (asterisk) was at 11 o’clock (**D**, **E**). Successful biliary cannulation finally achieved (**F**) (white arrowhead indicating guidewire was inside of common bile duct)

Difficult cannulation was encountered in 10 patients (Additional file 1: Table S2). Patient 1 and 2 was placed in prone or supine position during cannulation, trans-pancreatic precut were used as salvage method and the biliary orifice was then successfully exposed in the 3–5 o’clock direction (Fig. 2). Cannulation in patient 3 succussed after position changed from supine to prone. Patient 4 and patient 5 were placed in prone position or left lateral position, deep biliary cannulations were finally failed, and second-day attempts were denied because of complication or patient unwillingness. Patient 6 was placed in left lateral, while patient 7–10 were placed in supine position, all had achieved successful cannulation after persistent wire-guided cannulation.

In total, successful selective biliary cannulation was achieved in 85.7% (12/14), transpancreatic precut was used in 2 patients as a salvage method when standard cannulation failed. Needle knife precut was used in 1 patient as the initial cannulation method. No additional assistant skills were needed to facilitate papilla exposure or cannulation. The median operating time was 45 (27.5–60) minutes. Papillary sphincterotomy was successfully performed in 85.7% (12/14) patients. Other manipulation included large balloon dilation in 42.8% (6/14) of patients, basket retrieval of stones in 64.3% (9/14), balloon sweeping in 71.4% (10/14) and naso-biliary drainage in 42.8% (6/14). 1 patient was found to have mild benign hilar stricture during procedure and treated with 7Fr-10 cm plastic stent. Technical success rate was 85.7% (12/14). 3 patients received rectal indomethacin.

### Adverse events

The incidence of overall adverse events was 28.6% (4/14) (Table 3). Post-sphincterotomy bleeding occurred in 1 patient who received trans-pancreatic precut, which was successful treated by submucosal injection of epinephrine. 1 patient with chronic obstructive pulmonary disease developed pneumonia and 1 with coronary heart disease developed acute myocardial infarction. Both patients recovered with conservation treatment. Neither PEP nor perforation occurred. No death was found.

**Table 3:** Adverse events

	Patient (n = 14)	Grade of adverse event
Total complications, n (%)	3 (21.4)	
PEP	0	
Bleeding	1	mild
Perforation	0	
Infection	1	moderate
Myocardial infarction	1	moderate

*PEP*, post-ERCP pancreatitis

## Discussion

The present study presented ERCP outcomes of 14 SIT patients. Although the sample size was still small, to our knowledge, it represented the largest study concerning SIT patients who underwent ERCP to date. The study found that selective cannulation in SIT patients was difficult (71.4%, 10/14) even by experienced endoscopists. A final successful cannulation rate of 87.5% could be achieved without an increase in the incidence of PEP. Traditional position seemed to be more challenging for selective cannulation and procedural safety.

At present no consensus achieved on “standard position” for SIT patients undergoing ERCP. We searched the PubMed using terms “(situs inversus OR situs inversus totalis OR situs inversus viscerum) AND ERCP”. Totally 46 case reports with 49 patients were identified, including 41 patients with procedure details reported (summarized in Additional file 1: Table S3). Nearly half of patients were placed in usual prone position were, concerning the benefit of no need to change the endoscopic room setting and the position of endoscopists [6, 14]. Rocha M et al. reported that easier and more effective procedure in SIT patient under supine position because the organs disposition was similar to that of patients without situs inversus [8]. Right lateral position with mirror technique also reported in several studies which found that cannulation and sphincterotomy could be performed by classic maneuvers [7, 15, 16]. The patient-endoscopist placement and corresponding endoscopic room setup was at the endoscopist’s choice in our study. In the present study, more than 3/4 of patients were placed in modified position (i.e., supine, right or left lateral), had no advantage in terms of difficult cannulation (72.7% vs. 66.7%) compared to patients under traditional prone position, but numerically higher successful cannulation (90.9% vs. 66.7%) and less adverse events (18.2% vs. 33.3%) were observed (Table 4). Comparison of ERCP performed in situs solitus (normal) patient under prone and supine position had been reported by 2 studies, which indicated the tendency of increasing difficulty in the group of supine position even when endoscopists were experienced in reversed scope maneuver [17, 18]. Further large sample or prospective studies are needed to draw a conclusion on the optimal position of patient.

**Table 4:** Comparation of outcomes and complications of ERCP between SIT patients under different positions

Patient position	Modified position* N = 11	Prone N = 3	*P* value
Difficult cannulation, n (%)	8 (72.7)	2 (66.7)	1
Successful cannulation, n (%)	10 (90.9)	2 (66.7)	0.40
Technical success, n (%)	10 (90.9)	2 (66.7)	0.40
Procedure time(min), mean ± SD	41.4 ± 22.8	50 ± 17.3	0.56
Adverse events, n (%)	2 (18.2)	1 (33.3)	1

*Including supine, left or right lateral positions

Cannulation in SIT patient is considered difficult due to several aspects: 1.The direction of biliary orifice is inversed to 1–3 o’clock (Fig. 2d, e), thus selective biliary cannulation maneuvers should be performed inversely as per normal procedures [19, 20]. Inadvertent pancreatic duct (PD) cannulation might increase. Rotatable sphincterotome is proved to be useful in several studies [5, 9, 21, 22]; 2. Scope shortening in the duodenum may be more difficult when the scope had to be rotated through 180°. Better visualization or cannulation angle sometimes only achieved under long scope status at the cost of increased instability. 3. The variation in the anatomy of papilla may add more difficulty to papilla exposure and cannulation, i.e., PAD or ectopic papilla. As in our experience, it reasonable to apply DWT or transpancreatic precut in the early stage to rescue difficult cannulation, especially when inadvertent PD cannulation occurs.

It’s unclear whether the SIT condition would increase overall difficulty of ERCP procedure or not. The mean procedure time of SIT patients in our study seems longer than that of situs solitus patients in previous reports (43 min vs. 25–36 min) [17, 18, 23]. However, all 12 cases with access to targeted duct in our study achieved technical success within one-time ERCP procedure. The degree of procedural difficulty was limited to grade I–II in our study, as all the cases were uncomplicated CBDS. More complicated ERCP procedures were reported by several published SIT cases, such as repeated mechanical lithotripsy or spyglass-guided laser lithotripsy, or CBDS removal in case of B-II gastrectomy [24–26]. Laparoscopic surgery, or percutaneous drainage with or without rendezvous method were reported to rescue failed ERCP cases [27].

Complications of ERCP in SIT patients were rare according to published cases. Lakhtakia S et al. reported that one case of bleeding from portal biliopathy during stone removal was successfully treated by self-expandable metal stent and balloon compression [28]. Our post-sphincterotomy bleeding event occurred to a patient with decompensated cirrhosis, the cutting direction was at the opposite side (1–3 o’clock) as compare to usual condition (10–12 o’clock). Whether the vessel distribution around papilla in SIT condition also inversed is unknown. No PEP happened in this small series, though difficult cannulation and multiple inadvertently PD cannulation occurred in some cases. The uncommon cardiopulmonary adverse events happened in our series were both related to underlying disease.

The spectrum of pancreaticobiliary disease in SIT patients is not fully elucidated due to its rarity. The most common concomitant congenital disorder of SIT patients is cardiovascular abnormality, no congenital pancreaticobiliary disease in adult was reported until now [29]. The incidence of complete SIT in ERCP population is 0.02% (14/65838) in our study. Other than the common indication of CBDS, malignant biliary or pancreatic indications of ERCP were reported sporadically (Additional file 1: Table S1). More than half of case reports of ERCP performed in patients with SIT were published within the last 5 years, more case accumulation and retrospective study are needed to understand the pancreaticobiliary disease under SIT condition.

## Conclusions

In conclusion, SIT is a rare congenital condition of visceral left–right asymmetry disorder which imposes challenge on ERCP procedure. The present study found that higher cannulation difficulty and failure under SIT condition occurred even in the hands of ERCP experts. Though modified position of patients seems to bring benefits in easier manipulation and less complications, more confirmative data are needed. Detailed documentation of procedural parameters and complication will be important in developing standards of care. More case report and larger cohorts are needed to further characterize the experiences and outcomes of ERCP in patients with SIT.

## Supplementary Information

**Additional file 1**: Supplementary tables.

## Data Availability

The data that support the findings of this study are available on reasonable request from the corresponding author (Xiangping Wang, email: windxp2013@163.com).

## Abbreviations

CBDS: Common bile duct stones
CT: Computed tomography
DWT: Double-wire technique
ERCP: Endoscopic retrograde cholangiopancreatography
IQR: Interquartile range
MRI: Magnetic resonance imaging
PAD: Periampullary diverticulum
PD: Pancreatic duct
PEP: Post-ERCP pancreatitis
SIT: Situs inversus totalis
SD: Standard deviation
