# Supplementary material for: Efficacy and safety of ERCP in patients with situs inversus totalis: multicenter case series and literature review
Source: BMC Gastroenterol. 2022 Nov 30;22:497. doi: 10.1186/s12876-022-02593-3 (PMC9714160; doi:10.1186/s12876-022-02593-3)
Supplement: Supplementary file 1 — Additional file 1: Supplementary tables. [file 12876_2022_2593_MOESM1_ESM.docx]

Supplementary materials

Supplementary table 1. Definition of high-risk patients for post-ERCP pancreatitis (PEP)

Abbreviations: ERCP, endoscopic retrograde cholangiopancreatography.

| Patients were considered high-risk for PEP if they met at least one of the major criteria, or two or more of the minor criteria: | |
| --- | --- |
| Major criteria | - Clinical suspicion of sphincter of Oddi dysfunction - History of PEP - Pancreatic sphincterotomy - Precut sphincterotomy - ≥8 cannulation attempts - Pneumatic dilatation of an intact biliary sphincter - Papillectomy |
| Minor criteria | - Women younger than 50 years - History of recurrent pancreatitis (≥2 times) - ≥3 injections of contrast into the pancreatic duct with ≥1 injection to the tail of the pancreas - Opacification of pancreatic acini - Brush cytology performed on the pancreatic duct |

Supplementary table 2. Details of ERCP procedure in 14 patients with SIT

| Patient No. | Gender/  age  (years) | Comorbidities | ERCP indication | Patient’s postion  (initial/change) | Cannulation methods | Difficult cannulation | Successful cannulation | Procedures | Procedure time (min) | PEP preventions | Complications |
| --- | --- | --- | --- | --- | --- | --- | --- | --- | --- | --- | --- |
| 1 | F/71 | No | CBDS Cholangitis | Prone | Wire-guided, precut | Yes | Yes | EST, ENBD,  stone removal by basket | 60 | No | No |
| 2 | M/49 | Cirrhosis | CBDS | Supine | Wire-guided, precut | Yes | Yes | EST, stone removal by basket + balloon | 30 | rectal indomethacin | Bleeding |
| 3 | M/70 | HBP, CHD, COPD | CBDS Cholangitis | Supine/  To prone | Wire-guided | Yes | Yes | EST, EPLBD, stone removal by basket + balloon | 90 | rectal indomethacin | No |
| 4 | F/77 | T2DM, CHD | CBDS Cholangitis | Prone | Wire-guided | Yes | No | NA | 60 | No | Myocardial infarction |
| 5 | F/37 | No | BAP | Left lateral | Wire-guided | Yes | No | NA | 20 | rectal indomethacin | No |
| 6 | M/56 | No | CBDS | Left lateral | Wire-guided | Yes | Yes | EST, EPLBD,  stone removal by balloon | 60 | No | No |
| 7 | M/27 | No | CBDS | Supine | Wire-guided | Yes | Yes | EST, EPLBD, ENBD,  stone removal by basket + balloon | 40 | No | No |
| 8 | M/47 | No | CBDS Cholangitis | Supine | Wire-guided | Yes | Yes | EST, EPLBD, ENBD,  stone removal by basket + balloon | 50 | No | No |
| 9 | M/37 | No | CBDS Cholangitis | Right lateral/  to supine* | Wire-guided | Yes | Yes | EST, EPLBD, ENBD,  stone removal by basket + balloon | 50 | No | No |
| 10 | M/48 | No | CBDS | Supine | Wire-guided | Yes | Yes | EST, EPLBD, biliary stenting,  stone removal by basket + balloon | 50 | No | No |
| 11 | M/87 | HBP, CHD, COPD | CBDS Cholangitis | Left lateral | Needle knife precut | No | Yes | EST, ENBD,  stone removal by balloon | 15 | No | Pneumonia |
| 12 | F/42 | No | CBDS Cholangitis | Left lateral | Wire-guided | No | Yes | EST,ENBD,  stone removal by basket + balloon | 40 | No | No |
| 13 | M/70 | HBP, CHD | CBDS Cholangitis | right lateral | wire-guided | No | Yes | EST, ENBD, stone removal by basket | 10 | No | No |
| 14 | M/77 | No | CBDS | prone | wire-guided | No | Yes | EST, EPLBD, ENBD,  stone removal by balloon | 30 | No | No |

Abbreviations: BAP, biliary acute pancreatitis; CBDS, common bile duct stone; CHD, coronary heart disease; COPD, chronic obstructive pulmonary disease; ENBD, endoscopic naso-biliary drainage; EPLBD, endoscopic papillary large balloon dilation; ERCP, endoscopic retrograde cholangiopancreatography; EST, endoscopic sphincterotomy; HBP, high blood pressure; NA, not applicable; PEP, post-ERCP pancreatitis; SIT, situs versus totalis; T2DM, type 2 diabetes mellitus.

*Position changed for better papilla visualization before cannulation.

Supplementary table 3. Published cases of ERCP in patients with SIT

|  | n=41 |
| --- | --- |
| Age, mean±SD | 66.9±15.1 |
| Male, n(%) (n=39) | 21 (53.8) |
| ERCP indication, n(%) |  |
| CBDS | 24 (58.5) |
| Benign biliary stricture | 7 (17.1) |
| Malignant biliary stricture | 7 (17.1) |
| SOD | 1 (2.4) |
| Pancreatic disease | 1 (2.4) |
| Others | 1 (2.4) |
| Patient position, n(%) |  |
| Prone | 20 (48.8) |
| Left lateral | 9 (22.0) |
| Supine | 6 (14.6) |
| Right lateral | 6 (14.6) |
| Special sphincterotome, n(%) | 6 (14.6) |
| Rotatable | 4 |
| Long-nose | 1 |
| Needle knife | 1 |
| Cannulation success, n(%) | 41 (100) |
| Technical success, n(%) | 41 (100) |
| Complication, n(%) | 1 (0.02) |

Abbreviations: CBDS, common bile duct stone; ERCP, endoscopic retrograde cholangiopancreatography; SIT, situs versus totalis; SOD, sphincter of Oddi dysfunction.
